# Study of the Reinforcing Effect and Antibacterial Activity of Edible Films Based on a Mixture of Chitosan/Cassava Starch Filled with Bentonite Particles with Intercalated Ginger Essential Oil

**DOI:** 10.3390/polym16172531

**Published:** 2024-09-06

**Authors:** David Castro, Aleksandr Podshivalov, Alina Ponomareva, Anton Zhilenkov

**Affiliations:** 1 Center for Chemical Engineering, ITMO University, Kronverkskiy Prospekt, 49, 197101 Saint-Petersburg, Russia; dcastrovargas@itmo.ru (D.C.); ap_k@inbox.ru (A.P.); 2 Institute of Robotics and Intelligent Systems, Saint-Petersburg State Marine Technical University, Lotsmanskaya Str., 3, 190121 Saint-Petersburg, Russia; zhilenkovanton@gmail.com

**Keywords:** chitosan/starch films, ginger essential oil, bentonite microparticles, encapsulation, antibacterial filler, reinforcing filler

## Abstract

Edible films based on biopolymers are used to protect food from adverse environmental factors. However, their ample use may be hindered by some challenges to their mechanical and antimicrobial properties. Despite this, in most cases, increasing their mechanical properties and antibacterial activity remains a relevant challenge. To solve this problem, a possible option is to fill the biopolymer matrix of films with a functional filler that combines high reinforcing and antibacterial properties. In this work, biocomposite films based on a mixture of chitosan and cassava starch were filled with a hybrid filler in the form of bentonite clay particles loaded with ginger essential oil (GEO) in their structure with varied concentrations. For this purpose, GEO components were intercalated into bentonite clay interlayer space using a mechanical capture approach without using surface-active and toxic agents. The structure and loading efficiency of the essential oil in the obtained hybrid filler were analyzed by lyophilization and laser analysis of dispersions, ATR-FTIR spectroscopy, thermogravimetry, and X-ray diffraction analysis. The filled biocomposite films were analyzed using ATR-FTIR spectroscopy, optical and scanning electron spectroscopy, energy dispersive spectroscopy, mechanical analysis under tension, and the disk diffusion method for antibacterial activity. The results demonstrated that the tensile strength, Young’s modulus, elongation at the break, and the antibacterial effect of the films increased by 40%, 19%, 44%, and 23%, respectively, compared to unfilled film when the filler concentration was 0.5–1 wt.%.

## 1. Introduction

The production of edible films has notably increased in recent years. Edible films keep food protected and fresh and are used to preserve meat, fruit, and vegetables and extend their shelf life [1]. These biodegradable films preserve the water content, texture, color, and flavor of the foods. Proteins, lipids, and polysaccharides are some of the raw materials (biopolymers) used for edible films [2,3]. Starch-based biopolymers are cost-effective, abundant, edible, and biodegradable polysaccharides and, as such, are promising feedstock to obtain edible films [4,5]. Cassava starch is an abundant biopolymer obtained from the root of the cassava plant (*Manihot esculenta*) cultivated in tropical and subtropical regions of the world, including Asia, Africa, and South America. It is the third largest source of carbohydrates among vegetables and one of the most important crops world-wide, representing staple security for more than one billion people [6,7].

Films based on cassava starch have been used in the food industry, medicine, and other fields [8,9,10]. These films are colorless, odorless, tasteless, impermeable to oxygen, biodegradable, and edible. However, films based on cassava starch are hydrophilic and exhibit a relatively high degree of crystallinity. For this reason, biodegradable packaging made from bio-based films is highly sensitive to moisture and has a poor water barrier and low mechanical properties compared to packaging made of synthetic polymers. To address this challenge, researchers have produced a combination of starch-based edible films with other biopolymers, plasticizers, and reinforcing agents [11,12,13]. One of the biopolymers used to improve edible films based on cassava starch is chitosan, a widely available natural, non-toxic, biodegradable, and abundant polysaccharide obtained from chitin deacetylation. Chitosan also evinces antimicrobial activities due to its polycationic characteristics [14,15]. Edible films based on chitosan and starch are formed from the combination of their hydrogen bonding and the opposite charge attraction between the chitosan cations and the negatively charged one on the surface of the starch. This provides adequate adhesion between the starch and the chitosan, forming edible films that exhibit superior moisture barrier and water resistance properties, including dimensional stability, as well as antimicrobial activities provided by the chitosan [15,16,17].

Edible films based on chitosan and starch are used to eliminate or inhibit spoilage and microbial activity that contaminate food. However, these antimicrobial properties may vary depending on the pH values, molecular weight, and types of bacteria [15]. Therefore, various antibacterial agents of natural origin, including metal ions, metal oxides, plant extracts, and essential oils, are added to the edible films to improve their protective antibacterial characteristics [10,12,18]. Essential oils refer to substances extracted from aromatic plants. Some of them can be used in food preservation and protection, as well as for the inhibition of foodborne pathogens [19,20,21]. Among these essential oils, ginger essential oil (GEO) extracted from the roots of the *Zingiber officinale Roscoe* plant is notable for its antimicrobial, antifungal, and antioxidant properties [22,23,24]. The addition of GEO in films based on chitosan and starch increases the antibacterial properties of the biopolymer films, resulting in the extended shelf life of food [15,23,25]. While the addition of essential oils to the biopolymer film is essentially beneficial, their direct incorporation into the film-forming solution may be a disadvantage due to their volatile nature, as they can evaporate and degrade in a short time, causing a reduction in the mechanical properties of the biocomposite films. One of the options to address this issue is the encapsulation of the essential oil in a multilayer structure, such as that presented by clays [26,27,28].

Clays, such as bentonites, can be added to the polymer matrix and serve as reinforcing components, improving the functional properties of the biopolymer films, including the gas and water vapor barrier, mechanical strength, and thermic and rheological properties; moreover, bentonites can be used as a filler for food packaging applications [13,29,30,31,32]. Clays are abundant, low-cost, environmentally friendly, and non-toxic materials with properties such as a large surface area, porosity, layer spacing, and thermal and chemical stability. Due to these properties, they can be used for the absorption and protection of essential oils [33]. Encapsulation is an effective method to protect the quality and improve the delivery systems of temperature-sensitive substances, such as essential oils. Reducing evaporation or slowing the mass transfer of volatile compounds to the external environment are some of the advantages of loading the essential oil into the silicate. This procedure also facilitates the application of essential oils in the polymeric matrix by transforming them into a solid phase, providing their control and targeted release, increasing shelf life, and masking flavors or odors [28,34]. Microemulsions, the use of surfactants and synthetic toxic agents, high-pressure homogenization, high shear homogenization, and ultrasonication are some of the techniques used for loading the essential oil into the clays, but only mechanical methods, like stirring or ultrasonication, lead to the use of clays for edible purposes [28,34,35].

The main aim of the present study was to obtain biocomposite films based on chitosan and cassava starch via the casting method filled with a reinforcing and antibacterial hybrid filler, such as bentonite clay/GEO particles, to simultaneously increase both the mechanical strength and antibacterial resistance of the films. For this purpose, bentonite clay/GEO hybrid filler particles were obtained by activating the interlayer space of bentonite clay microparticles with subsequent intercalation (loading) of GEO components into the interlayer space of clay particles by direct mechanical capture from an oil-in-water microemulsion. The application of the hybrid filler creation approach is due to the challenge of suppressing degradation processes in essential oil, increasing adhesion between the polymer matrix and the filler containing essential oil, and reinforcing the polymer matrix of films in the presence of essential oil. The efficiency of loading GEO components into the structure of bentonite clay particles, as well as the structure of dispersions of the obtained filler, were established by lyophilization and laser analysis of dispersions, ATR-FTIR spectroscopy, thermogravimetry, and X-ray diffraction. The effectiveness of reinforcing the polymer matrix of the film based on chitosan and cassava starch, as well as an increase in antibacterial properties when filled with a hybrid filler, was demonstrated.

## 2. Materials and Methods

### 2.1. Materials

Cassava starch (Thai Food King, Samutprakarn, Thailand) and chitosan (Bioprogress, Biokombinata, Russia) were used as biopolymers to obtain film-forming solutions and films. Sorbitol (Sigma-Aldrich, Saint Louis, MO, USA) was used as a plasticizer. Nanoclay, hydrophilic natrium bentonite CAS# 1302-78-9 (Sigma Aldrich, Saint Louis, MO, USA) was used as a reinforcing agent, and ginger essential oil (GEO) (Oleos, Moscow, Russia) was used as an antimicrobial agent. Distilled water and ethanol were used as solvents and media for making filler dispersions.

### 2.2. Preparation of Bentonite Clay/GEO Particles Dispersions and Its Characterization

The preparation of bentonite clay/GEO particle dispersion was divided into three steps. The first stage of the process was the clay swelling. For that, a suspension of 3 wt.% was prepared by adding bentonite clay to distilled water. The suspension was stirred for 48 h with a constant temperature of 60 °C. A constant temperature with an accuracy of ± 0.2 °C was maintained using a thermostatic thermometer ETS–D6 (IKA–Werke, Staufen, Germany). The second stage consists of the ultrasonic treatment. At first, the suspension was sonicated in an ultrasonic bath S 60 H (Elma, Czestochowa, Poland) at 37 KHz frequency for 15 min; then, the suspension was stirred in the ultrasonic disperser for 30 min at room temperature. After that, a solution of GEO in ethanol 2.24 vol.% (75 wt.% of mass of bentonite particles) was added with constant stirring for 24 h at 25 °C. Then, the dispersion of clay and essential oil were mixed at 8000 rpm on a mechanical disperser Ultra-Turrax T 25 digital (IKA–Werke, Staufen, Germany) for 30 min at 25 °C. Posterior obtained dispersion was fractionated by centrifugation at 3500 rpm for 15–20 min using a centrifuge CM-6M (Elmi, Saint-Petersburg, Russia), and the middle fraction was separated and cleaned with some cycles mixing water and centrifugation to remove not encapsulated essential oil. Finally, the cleaned fraction was lyophilized at a constant temperature of −35 °C and vacuum pressure of 1.65 mPa for 72 h in a Triad freeze dryer (Labconco, MO, USA).

For the characterization of the bentonite clay/GEO particle dispersion, the particle size distributions of the suspension after each stage were obtained using the laser analyzer Analysette 22 Nano Tec plus (Fritsch, Feldkirchen, Germany). The efficiency of the loading of GEO into the bentonite clay particles was analyzed using the lyophilization technique. The chemical structure of each separated fraction after lyophilization and of the GEO sample were determined using an FTIR spectrometer Tensor 37 (Bruker, Karlsruhe, Germany) equipped with the ATR unit MIRacle (Pike, WI, USA) with a crystal of ZnSe within the 4000–500 cm^−1^ range of wavenumbers. The thermal stability of each separated fraction of the filler dispersion and of the GEO was determined using thermomicrobalance TG 209 F1 Libra (Netzsch, Selb, Germany) in a dry nitrogen atmosphere with a constant heating rate of 10 °C/min in a temperature range of 25 to 900 °C, and alumina oxide crucibles (Al_2_O_3_) were used as sample carriers. For a more detailed understanding of the filler structure, lyophilized samples of bentonite clay particles activated in water and separated middle fraction of bentonite clay/GEO particles dispersion were analyzed for their X-ray diffraction patterns using an Ultima IV diffractometer (Rigaku, Osaka, Japan) at an operating voltage of 35 kV, a current of 15 mA with CuKα radiation wavelength of *λ* = 1.5418 Å in the range of 2*θ* angle from 10 to 80. The d-spacing was calculated according to Bragg’s law [36]:

(1) $$d=\frac{n\lambda }{2\sin\theta }$$

where n is an integer, and it is assumed to be 2; *λ* is the wavelength of the incident X-rays and 2*θ* is the diffraction angle.

### 2.3. Preparation of Films of a Mixture of Chitosan and Cassava Starch and Selection of a Composition for Obtaining Biocomposite Films

First, films of a mixture of chitosan and cassava starch in different dry mass ratios of biopolymers were obtained by casting a film-forming solution to select the polymer matrix of the biocomposite films. For this, the film-forming solutions were prepared by dissolving chitosan and cassava starch in distilled water with a final concentration of 2.5 wt.% of polymer with different ratios of starch and chitosan. Individual solutions of chitosan and cassava starch were stirred for 30 and 60 min at constant temperatures of 40 °C and 90 °C, respectively. After polymer dissolving, a series of film-forming solutions, chitosan/starch, were prepared by mixing them with different ratios from 100 to 100 wt.% chitosan and starch with an increment of 10 wt.%. Then, sorbitol in 25 wt.% from the dry mass of biopolymers was added to each chitosan/starch solution and mixed at a constant temperature of 60 °C for 30 min. After that, the casting technique was used to prepare the films. Prepared solutions of chitosan/starch in a volume of 15 mL were molded to Petri dishes with a diameter of 90 mm, followed by drying in a laboratory oven at 35 °C with constant air convection for 24 h. The obtained films were named as chitosan/starch ratio of solution volume.

**Figure 1:**
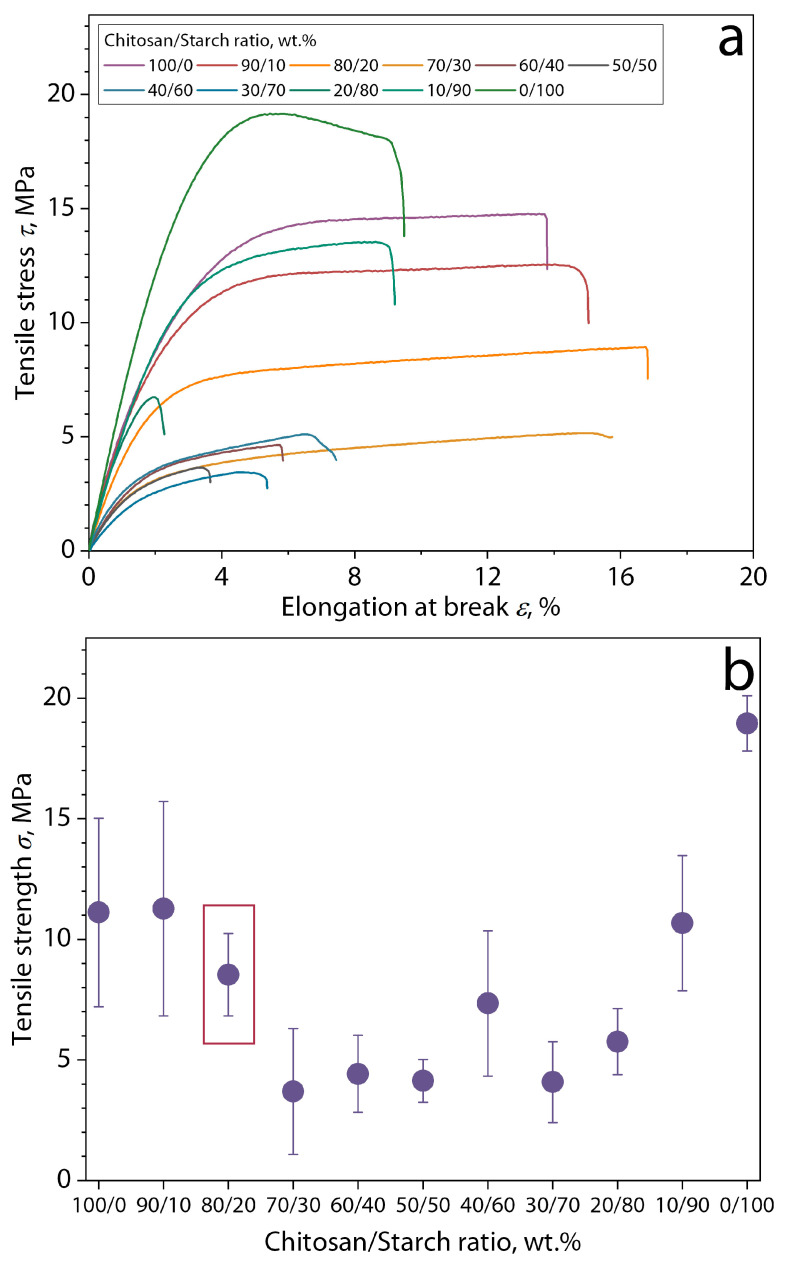
(**a**) Exemplary tensile curves and (**b**) tensile strength of chitosan/cassava starch films depending on the polymer’s ratio.

To select the composition of the biopolymer matrix and subsequently prepare the biocomposite, the obtained films of the chitosan/starch mixture with different polymer ratios were first subjected to mechanical analysis under tension using a universal testing machine, Instron 5943 (Instron, Norwood, MA, USA). For this purpose, film samples in the form of strips with dimensions of 50 × 10 mm, seven pieces for each composition, were subjected to tensile deformation at a rate of 15 mm/min at room temperature. The selection of the composition of the biopolymer matrix was based on two principles: (1) the closest ratio of components to 50/50 wt.% due to the chitosan is an effective and natural antimicrobial agent [15,16,17] and (2) the highest average tensile strength (Figure 1), essential parameter of edible films to assess the ability of the film to withstand external force and maintain the integrity of the film without breaking [37]. Thus, a chitosan/starch ratio of 80/20 wt.% was selected as the biopolymer matrix for obtaining filled biocomposite films.

**Figure 2:**
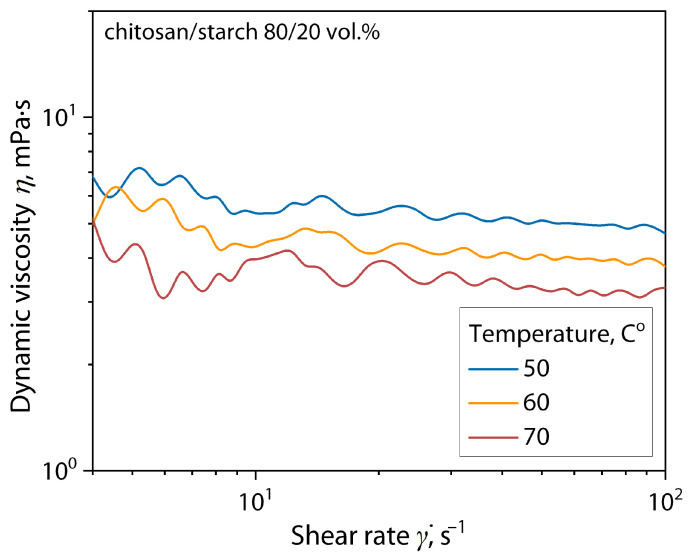
Flow curves of the film-forming solutions of chitosan/cassava starch mixture 80/20 wt.% at different temperatures.

Then, film-forming solutions of the chitosan/starch mixture 80/20 wt.% were subjected to dynamic viscometry to establish the effect of solution temperature on its viscosity. These preliminary studies are necessary to select the minimum possible solution temperature to prevent the degradation processes of the ginger essential oil (GEO) components and their evaporation and reduce their antibacterial activity. For this purpose, the dynamic viscosity of the film-forming solution (Figure 2) was analyzed using a Physica MCR 502 rotational rheometer (Anton Paar, Graz, Austria) equipped with a cylinder-in-cup measuring system with an external cylinder diameter of 26.66 mm and an internal cup diameter of 28.92 mm in the shear rate (γ̇) range from 0.1 to 100 s^−1^ at various constant solution temperatures of 50, 60 and 70 ± 0.2 °C.

The flow curves show that in the range of shear rate values, at which the film-forming solution is usually formed by casting onto a substrate, an almost constant coefficient of dynamic viscosity is observed for the solution at all temperatures. This behavior corresponds to Newton’s law of viscous friction and indicates the possibility of forming this solution by casting it onto a substrate without restrictions. When the solution temperature decreases from 70 to 50 °C, there is a slight increase in the viscosity of the solution (less than 10%), which cannot affect its formation but allows reducing the effect of degradation of the antibacterial properties of the essential oil when it is introduced into the solution. Therefore, a solution temperature of 50 °C was used to form films.

### 2.4. Preparation of Biocomposite Films of a Mixture of Chitosan and Cassava Starch Filled with Bentonite Clay/GEO Particles

For the obtention of the films with bentonite clay/GEO particles into the prepared individual chitosan solution, as described above, different concentrations of bentonite clay/GEO particles dispersion such as 0.5, 1, and 2 wt.% from the dry mass of biopolymer mix were added with constant stirring for 30 min at 40 °C. Next, a known volume of starch solution, prepared as described above, and sorbitol of 25 wt.% from the dry mass of biopolymers was added to each solution chitosan/filler dispersion and mixed at a constant temperature of 50 °C. After that, a casting technique was used to prepare the biocomposite films. Prepared solutions of chitosan/starch/filler dispersion in a volume of 15 mL were molded and dried as described above.

### 2.5. Characterization of the Biocomposite Films

For analyzing the morphology of the films, an optical microscope in transmission mode STM6 (Olympus, Tokyo, Japan) and a scanning electronic microscope Mira III (Tescan, Jihomoravsky, Czech Republic) working at 2.0 kV voltage were used. In addition, a secondary electron (SE) detector was used for scanning electron microscopy of films for energy dispersive spectroscopy (EDS) analysis and obtaining EDS maps of Si Kα1. Film thickness was determined by directly measuring 10 different points for each film with a digital micrometer (Tehrim, Saint-Petersburg, Russia) with an accuracy and precision of 0.004 mm. The composition of the biocomposite films was confirmed using the ATR-FTIR spectroscopy method for unfilled films. The tensile strength (*σ*), Young’s modulus (*E*), and elongation at break (*ε*) of films were measured as described above. For the antimicrobial activity, the disc diffusion method was used to evaluate the antimicrobial activity of the films against *Escherichia coli* and *Staphylococcus aureus* [38]. The film samples were cut in disc form with 5 mm of diameter and then collocated over the sterilized glass Petri dishes with 100 mm diameter, which contain agar and ∼108 CFU/mL (0.5 McFarland) of *S. aureus* and *E.coli*, independently. Then, the dishes were incubated at 35 °C for 74 h. Finally, the area of the inhibition was measured using ImageJ software 1.53t [25,38,39].

## 3. Results and Discussion

### 3.1. Characterization of the Loading Efficiency of GEO into the Bentonite Clay Particles

**Figure 3:**
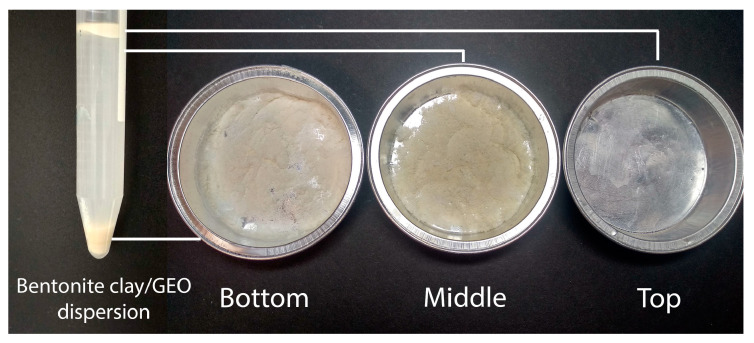
Bentonite clay/GEO particles dispersion in water after centrifugation and top, middle, and bottom separated fractions of the dispersion after lyophilization process.

Figure 3 shows a photograph of the bentonite clay/GEO particle dispersion in water after centrifugation and the separated individual fractions of dispersion after lyophilization.

Lyophilization of the separated individual fractions of the filler dispersion clay/GEO after fractionation made it possible to make a visual qualitative analysis of their composition. The top fraction, characterized by a white foam exhibiting a distinct aroma of essential oil, completely evaporated without leaving any residue. In this case, given the fact that the lyophilization procedure predominantly leads to the evaporation of only dissolved low-molecular liquid components, it can be concluded that this fraction contains only GEO components and water.

The bottom fraction after lyophilization was a solid layer of gray-beige material consisting of large coagulated particles of bentonite clay with the presence of surface erosion. Considering the large volume of the solid material after lyophilization and the impossibility of settling the GEO components by centrifugation due to their low density compared to water and bentonite clay particles, it can be assumed that this fraction contains predominantly clay particles.

The middle fraction in its original form was a stable aqueous dispersion of particles with a pronounced visual scattering of light, and after its lyophilization, a soft porous layer of white color, which indicates the presence of bentonite particles of small size and mass. The stability of the initial dispersion of the middle fraction was high and persisted for at least three weeks. These facts indicate that the middle fraction of the dispersion may contain low-mass bentonite clay particles with components of GEO intercalated into the interlayer space, having a density significantly lower than bentonite. In addition, this effect leads to a decrease in the total density of clay particles and, as a consequence, to a strong increase in the stability of the dispersion. Such structuring suggests that ginger essential oil, in this case, can act not only as a bioactive bactericidal or fungicidal agent but also as a structuring agent that increases the stability of the dispersion of filler particles. Increasing the stability of the filler dispersion is one of the key factors preventing the coagulation of filler particles when it is introduced into biopolymer-based film-forming solutions. The presence of essential oil components in the structure of bentonite particles was also further proved using thermogravimetric analysis and the attenuated total reflectance Fourier transform infrared (ATR–FTIR) spectroscopy.

**Figure 4:**
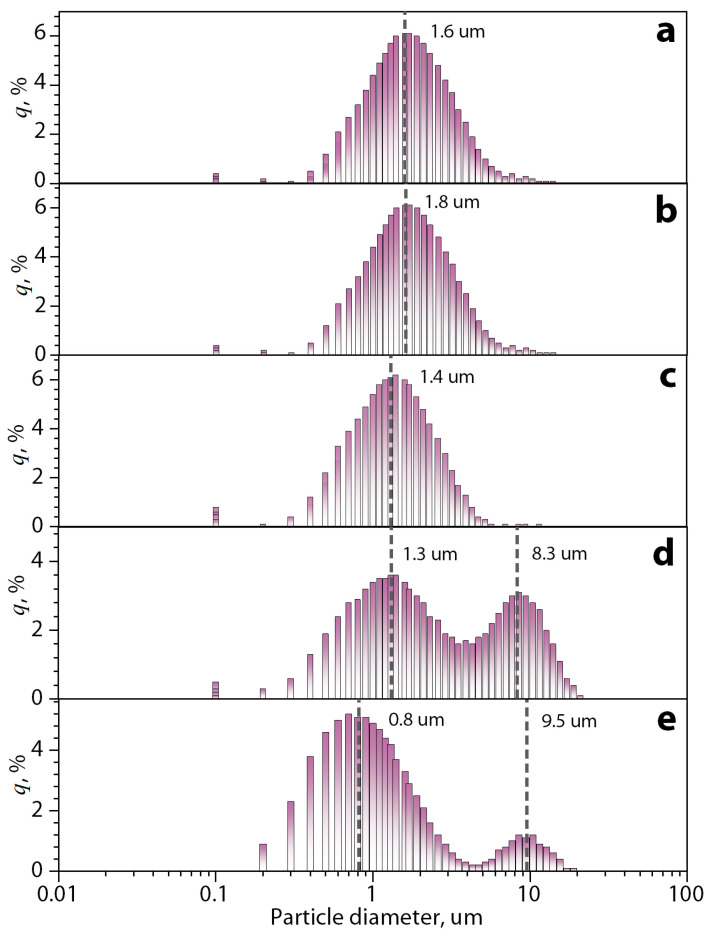
Particle diameter distributions for the (**a**) initial bentonite clay dispersion in water, (**b**) dispersion after swelling, (**c**) dispersion after swelling and ultrasonic treatment, (**d**) dispersion after swelling, ultrasonic and mechanical treatment, and (**e**) final dispersion with loaded of GEO.

The particle size distributions of the dispersion of bentonite clay particles after each stage of treatment are shown in Figure 4.

The initial average size of clay particles in water is about 1.6 μm (Figure 4a). After the swelling of the clay, the distance between the layers increases due to the interaction of water molecules and their hydrogen interaction with –OH groups on the surface of the layers in the interlayer space. However, the average diameter of the particles did not significantly change (1.8 μm) (Figure 4b). After ultrasonic treatment, the average diameter of the particles is reduced (1.4 μm) (Figure 4c). This effect is likely to have been achieved by the possible exfoliation of clay layers and their stacks by the effect of cavitation and air microblasts during ultrasonic treatment. The mechanical treatment of clay particle dispersions with the addition of a ginger essential oil solution in ethanol has made the distribution of objects bimodal distribution with an average size of 1.3 μm and 8.3 μm for the clay with similar cumulative distribution (q, %) (Figure 4d). It can be concluded that in this case, under a strong mechanical action on the layered clay structures during processing, two processes took place in parallel: the destruction of the original clay particles and their exfoliation with the formation of a fine fraction and microemulsion of the essential oil, the process of intercalation of essential oil components into the interlayer space of bentonite particles increasing their size and forming a second mode with a large average diameter of objects. On the other hand, the bimodal distribution persists, but with variation in both the mean clay sizes of 0.8 μm and 9.5 μm (Figure 4e), as well as in the cumulative distribution (q, %), being larger for smaller particles. This is because the fractionation process separates the clay particles that contain the GEO and forms a dispersion with the water from the clay particles with a large amount of the GEO and the essential oil that could not be encapsulated.

**Figure 5:**
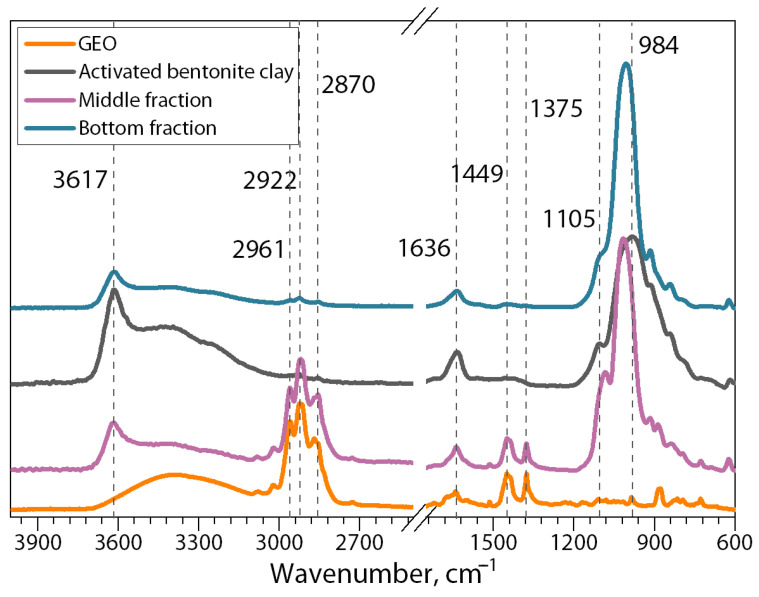
ATR-FTIR spectra of the GEO sample, bentonite clay particles activated by swelling in water and separated fractions of the dispersion of bentonite clay/GEO particles.

Figure 5 shows the ATR-FTIR spectra of GEO and bentonite clay particles after their activation in water and the middle and bottom separated fractions of the dispersion of bentonite clay/GEO particles.

On the spectrum of the GEO sample, the first region between the bands at 3700–3000 cm^−1^ represents the hydroxyl group stretching. The band at 2922 cm^−1^ corresponds to C-H stretching in alkanes. Aldehyde carbonyl C=O stretching vibration is represented by the band about 1636 cm^−1^. The vibration of the benzene ring skeleton is shown by the band at 1514 cm^−1^. C–O–H stretching of other phenolic compounds is represented by the band at 1126 cm^−1^. Finally, the phenyl group of cinnamaldehyde, distinctive of GEO, is represented by the peak at 1449 cm^−1^ [40,41,42].

The spectrum of raw bentonite clay after swelling shows a peak at 3617 cm^−1^ corresponding to the stretching vibrations of hydroxyl groups coordinated to the octahedral cation. The results of the stretching and bending vibrations of –OH functionality of absorbed water molecules on the clay nanolayer’s surface are shown by the band at 1636 cm^−1^. The Si–O stretching vibrations show the most intensive absorption peak at 984 cm^−1^.

In turn, the spectrum of the middle fraction of the bentonite clay/GEO particles dispersion after lyophilization demonstrates absorption in bands 2922 cm^−1^, 1514 cm^−1^, and 1449 cm^−1^ characteristics of the spectrum of the initial GEO sample [43,44]. This fact clearly indicates the presence of intercalated GEO components in the interlayer space of bentonite particles, given that GEO effectively evaporates during lyophilization. On the other hand, the spectrum of the bottom fraction shows the same peaks as the clay spectrum after swelling but with lower intensity, indicating the absence of GEO in this sample, thus demonstrating the efficiency of the GEO-loading in bentonite clay particles.

**Figure 6:**
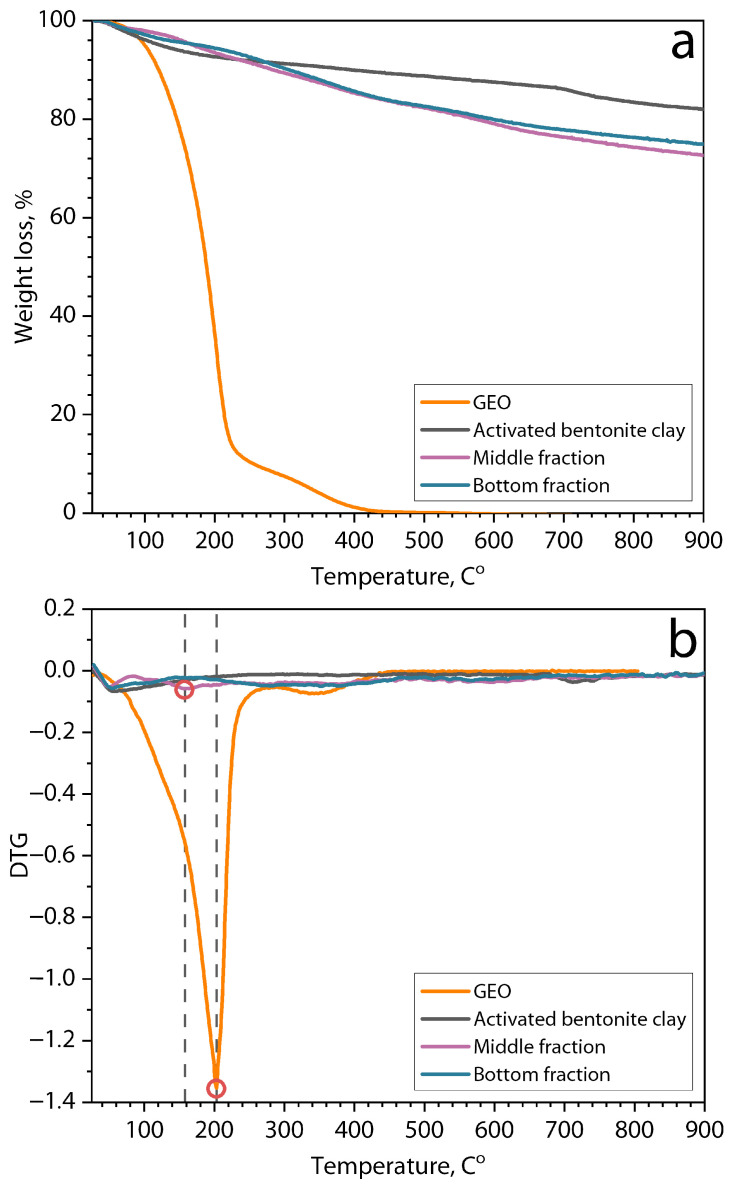
(**a**) Thermograms and (**b**) derivative thermogravimetric curves of weight loss of GEO sample, bentonite clay particles after their activation by swelling in water, and middle and bottom separated fractions of the dispersion of bentonite clay/GEO particles.

Figure 6 shows thermograms and the derivative thermogravimetric curves of weight loss of GEO sample, bentonite clay particles after their activation in water, middle, and bottom separated fractions of the dispersion of bentonite clay/GEO particles during their heating.

In the thermograms of all samples (Figure 6a), the observed first step of weight loss in the region of 100 °C is obviously associated with the evaporation of residual moisture. The thermogram of the original GEO sample demonstrates the second sharp step of weight loss by more than 85% with the center at 203 °C. This strong effect is associated with the low thermal stability of the GEO components, which leads to their thermal destruction [45,46]. On the other hand, the sample of bentonite clay particles activated in water demonstrates high thermal stability in the entire temperature range and a weight loss of only 8% when heated to 900 °C. Thermograms of the separated middle and bottom fractions of bentonite clay/GEO particle dispersions after lyophilization showed a large weight loss of 17.4 and 15%, respectively, when heated to 900 °C. Moreover, it is evident that the weight loss of these samples occurs almost without sharp steps, even at high temperatures. These observations may indirectly indicate the presence of intercalated GEO components, especially in the middle fraction (more weight loss), in the interlayer space of bentonite particles, and, consequently, the efficiency of their loading. On the other hand, the lower weight loss of the bottom fraction demonstrates that this fraction contains predominantly clay particles [44,47,48,49]. In addition, in the DTG curves of the middle fraction, a second peak in the temperature range of 160 °C was observed. Considering that in this range, there was no weight loss for the bentonite clay, but this peak may be attributed to the essential oil evaporation [50].

**Figure 7:**
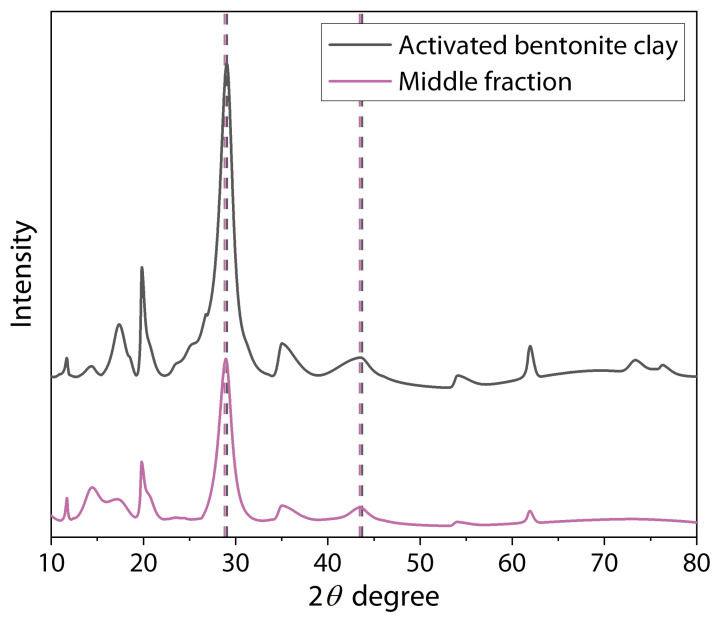
The XRD diffractograms of bentonite clay particles after their activation in water and middle fraction of the dispersion of bentonite clay/GEO particles.

The X-ray diffractograms for the bentonite clay particles after their activation in water and the middle fraction of the dispersion of bentonite clay/GEO particles are shown in Figure 7, and the basal d-spacing values are recapped in Table 1.

As shown in other works, characteristic peaks were observed in the diffraction patterns of bentonite at 2*θ* angle values of 20.7° (d = 4.36 Å), 36.3° (d = 2.60 Å), 54.7° (d = 1.88 Å) (JCPDS file card number 01-088-0891) [44,51] and of montmorillonite at 29.86° (d = 3.09 Å) and 45.57° (d = 2.15 Å) (card number 00-12-0219 Quality I). Comparing these values with those obtained for the samples of the middle fraction of the dispersion of bentonite clay/GEO particles, a slight shift in the peak position was observed. The Δ d-spacing values showed that the interlayer spaces increased by about 3 and 5%. This effect confirms the success of clay particle activation by physical methods (mechanical and ultrasound treatment) and obtaining a bigger space between layers (increase in the d-spacing) due to the cavitation effect [52,53,54]. In these cases, the changes are due to the water or small organic molecules of the GEO components penetrating into the clay structure, increasing the distance between layers and landing in the formation of intercalated clay filler [55,56,57].

### 3.2. Characterization of Chitosan/Cassava Starch Biocomposite Films Filled with Bentonite Clay/GEO Particles

**Figure 8:**
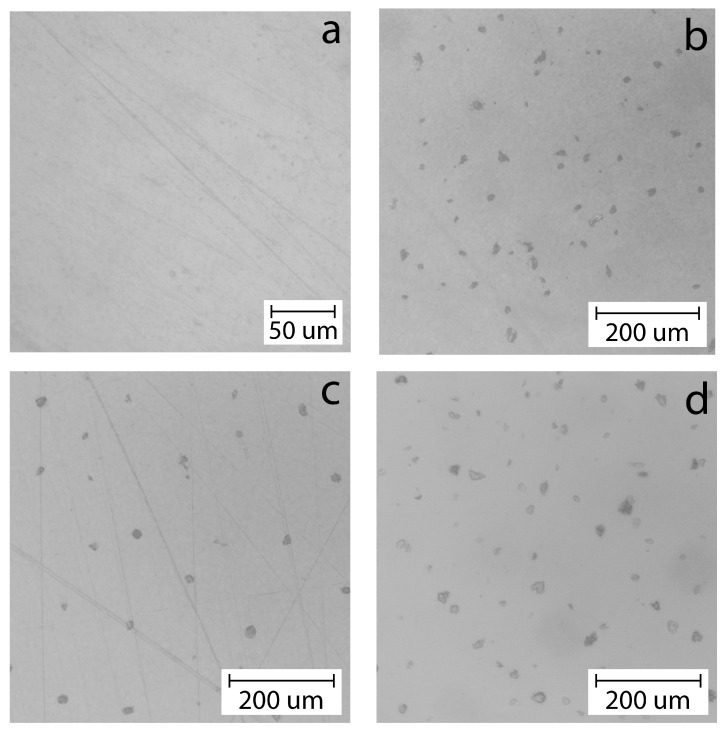
Optical microphotographs of biocomposite films based on chitosan/cassava starch 80/20 wt.% (**a**) unfilled and filled with (**b**) 0.5, (**c**) 1, and (**d**) 2 wt.% of bentonite clay/GEO particles.

Figure 8 shows optical micrographs of films based on a mixture of chitosan and cassava starch in a ratio of 80/20 wt.% dry base, as well as filled with 0.5, 1, and 2 wt.% bentonite clay/GEO particles.

Figure 8a shows that the initial polymer matrix based on biopolymers does not have a developed morphology, does not contain various defects and artifacts of the microstructure, and is also transparent to visible light. At this level of microstructure and the indicated ratio of biopolymers, their high miscibility and compatibility are observed, which leads to a completely isotropic structure.

On the other hand, the micrographs of biocomposite films filled with bentonite clay/GEO particles demonstrate dark areas that are opaque to visible light in the film structure and are associated with filler particles. On average, the diameter of these particles is about 20 μm. It is evident that these particles are almost equidistant from each other and well dispersed in the polymer matrix. Such effective dispersion can lead to the formation of a reinforcing network of filler particles and an increase in the mechanical properties of the original film. As was shown earlier by the results of the analysis of the structure of bentonite clay/GEO particles dispersions in water after their production, they contain two fractions with an average diameter of up to 1 μm and with diameters of 5–20 μm. Taking into account the limitations of the optical microscopy method in terms of maximum magnification, it can be assumed that only large particles included in the second statistical ensemble in the dispersion can be observed in these micrographs, while a large number of small particles in the film structure cannot be observed. For this reason, for a more detailed analysis of the microstructure of the films, they were subjected to scanning electron microscopy with increased resolution and high image contrast.

**Figure 9:**
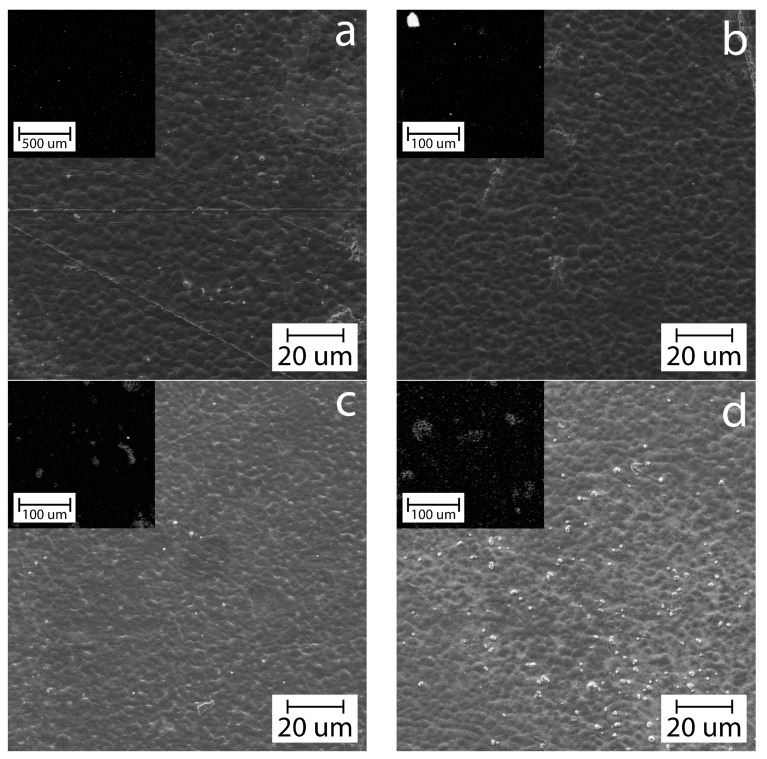
SEM microphotographs and EDS maps of silicon (in left upper corner) of biocomposite films based on chitosan/cassava starch 80/20 wt.% (**a**) unfilled and filled with (**b**) 0.5, (**c**) 1, and (**d**) 2 wt.% of bentonite clay/GEO particles.

Figure 9 shows SEM electron microphotographs and EDS maps of silicon (in the left upper corner) of chitosan/cassava starch unfilled and filled films with bentonite clay/GEO particles.

The EDS maps of silicon in the figures at low magnification clearly show the presence of large bentonite clay/GEO particles in the structure of the filled biocomposite films shown in light gray, especially at a filler content of φ > 0.5 wt.%. The SEM micrograph of the native unfilled polymer matrix Figure 8a demonstrates a dark, continuous granular film surface. We believe that this morphology is associated with limited thermodynamic mixing of the polymers due to their different crystallization rates during the drying of the films. Despite this, in general, the continuity of this structure at the micro level allows us to talk about the homogeneity of the films and the absence of the effect of coalescence of the phases of individual polymers. Electron micrographs of films containing filler show that as the filler concentration increases to >1 wt.%, small filler particles (white dots) with a diameter of <2 μm are present in the structure, and their number increases as the concentration increases. These observations are fully consistent with and confirm the results of the analysis of the structure of the dispersions of filler particles by laser analysis and optical microscopy of biocomposite films. These small particles are most likely embedded in the polymer matrix between large filler particles and can also participate in the formation of the reinforcing network of the filler, increasing the efficiency of reinforcement [40,58,59,60]. In addition, the optical and SEM micrographs of the films show a good distribution and absence of the particle agglomeration sites due to the small size of the filler particles and the concentration in the biopolymeric matrix. The larger particles and their high concentration can lead to the aggregation of the filler phase, which will precipitate at the bottom of the biocomposite [61,62]. The formation of such aggregates and sedimentation of the filler phase lead to an allotropic distribution of the filler in the polymer matrix of the finished film and significantly reduces the specific area of the polymer matrix-filler interface region, which leads to disruption of the reinforcing network of the filler and reduces the effectiveness of reinforcement [61,62,63].

**Figure 10:**
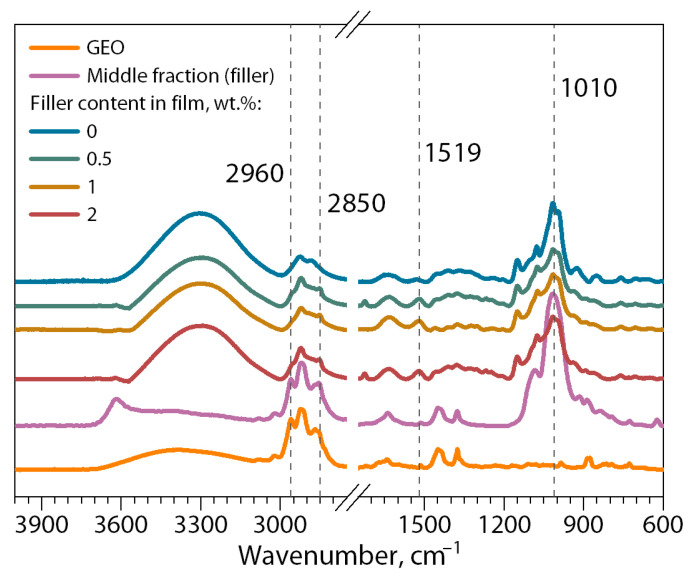
ATR-FTIR spectra of biocomposite films based on chitosan/cassava starch 80/20 wt.% unfilled and filled with bentonite clay/GEO particles.

Figure 10 shows the spectra for GEO, the middle separated fraction of the dispersion of bentonite clay/GEO particles (filler), and biocomposite films without and with the addition of filler.

In general, the ATR-FTIR spectra of the biocomposite films derived from chitosan and cassava starch filled with the dispersion of bentonite clay/GEO particles demonstrate similarities, revealing characteristic vibration bands attributed to biocomposite films obtained from starch and chitosan without filler. This observation suggests that the incorporation of the filler did not lead to the formation of novel covalent bonds. The peaks characteristic of starch structures (C–C, C–O– and C–H) in the range 1010–1100 cm^−1^ are clearly visible. Absorption bands of 1519–1529 cm^−1^ corresponding to chitosan amino groups are also observed. In the region of 3000–3600 cm^−1^, valence oscillations of O–H and N–H are traditionally observed. In spite of that, filled biocomposite films present more intense absorption at some bands in the region of 2850–2960 cm^−1^, and 1377 cm^−1^ correspond to C–H stretching for an alkane, aldehyde carbonyl C=O stretching vibration, and the vibration of the benzene ring skeleton present in the GEO composition. These results show that the filled biocomposite films contain both chitosan and cassava starch and bentonite clay/GEO filler particles, and no chemical interactions occur between these components [64,65].

**Figure 11:**
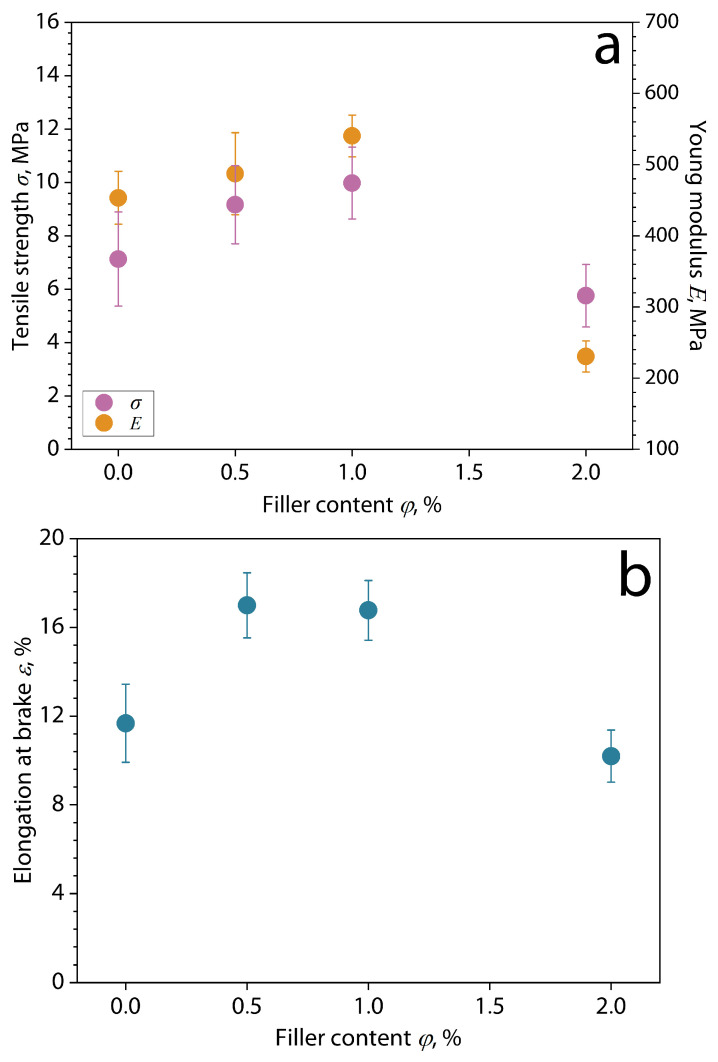
(**a**) Tensile strength, Young’s modulus and (**b**) elongation at break of biocomposite films based on chitosan/cassava starch 80/20 wt.% filled with bentonite clay/GEO particles depending on the filler content.

The reinforcing effects of the filler addition on the mechanical properties of biocomposite films at tension are presented in Figure 11.

The results demonstrate that the tensile strength, Young’s modulus, and elongation at the break of the films increase with the addition of bentonite clay/GEO filler particles at concentrations of *φ* = 0.5–1 wt.%. Inversely, the higher concentration of the dispersion (*φ* = 2 wt.%) reduced these parameters. This effect is probably a consequence of the formation of an effective reinforcing network of filler particles in the polymer matrix, as shown above by the results of optical microscopy and SEM of biocomposite films. This strengthening is also associated with the effective dispersion of filler particles in the polymer matrix and high adhesion between chitosan and starch macromolecules and the surface of bentonite particles due to hydrogen interactions [10,11,16,31]. Studies have reported that hydrogen bonds can be formed by interaction between the filler particles with the hydroxyl groups of biopolymers, leading to the strengthening of molecular forces. This effect helps maintain the structural integrity of the polymer matrix under stress [66,67,68]. Despite this, this effect is a threshold, and exceeding the filler concentration threshold of *φ* = 2 wt.% probably leads to overloading of the matrix with filler and the formation of stress areas, which disrupt the filler interaction with the biopolymer matrix, generating poor dispersion and inadequate bonding [69,70,71]. It is clear that the strengthening of the polymer matrix occurs despite the presence of intercalated components of ginger essential oil (GEO) in the structure of the filler particles. The influence of the incorporation of essential oil into cassava starch films without encapsulation or intercalation was studied by Utami et al. [72]. They found that increasing the concentration of the essential oil decreases the mechanical properties of the films because the addition of essential oil without encapsulation induces a heterogeneous film structure, which is the reason for the reduced mechanical properties [17]. In contrast, the encapsulation of the essential oil causes an increase in these properties [39].

**Figure 12:**
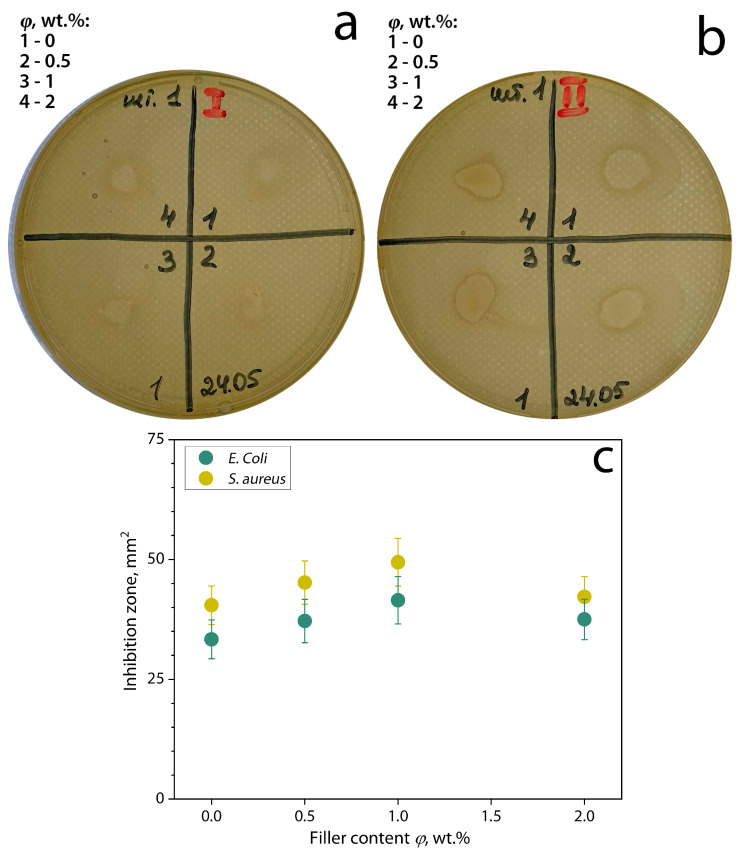
Example of diffusion-disc method on the biocomposite films samples based on chitosan/cassava starch 80/20 wt.% filled with bentonite clay/GEO particles against (**a**) *E. coli*, (**b**) against *S. aeurus* and (**c**) inhibition zone depending on the filler content.

The antimicrobial activity of the biocomposite films is shown in Figure 12.

The results showed that the introduction of bentonite clay/GEO filler particles into the polymer matrix increases the antimicrobial activity properties of the film. This is due to the fact that the essential oil is inside the filler particles and can be released in a controlled form, as an effect of encapsulation, by controlled diffusion [28,73]. Then, the essential oil moves into the polymeric matrix, providing antimicrobial activity to the biocomposites. The antimicrobial activity of the films is due to the interaction between the essential oil and the bacterial wall [74]. Falleh et al. [20] mentioned that this interaction causes damage to the cell wall, degradation of the cell membrane, coagulation of the cytoplasmic protein, leakage of the cell contents, and a reduction in the proton-motive. Also, it is shown that *E. coli* is less susceptible than *S.aureus* to GEO. Several studies also confirmed the susceptibility of Gram-positive bacteria, such as *S. aureus*, in comparison with Gram-negative bacteria *E. coli* [19,23,74,75,76,77,78]. In this respect, a thick outer lipopolysaccharide layer covers the *E. coli* surface, providing extra protection against antimicrobial agents, while only a monopeptide layer structure covers the surface of *S. aureus* [74,79,80]. On the other hand, the encapsulation of the essential oil allows its controlled release within the film, as well as protecting it from evaporation and oxidization when the film is obtained and dried [19,25,38,64,81]. However, a large amount of filler at *φ* = 2 wt.% can lead to a decrease in this effect because the particles can overlap each other, inhibiting the release of GEO.

## 4. Conclusions

In the present work, films based on chitosan and cassava starch were obtained by the casting method of a film-forming solution without and with variable concentrations of bentonite clay/GEO filler particles (0.5, 1, and 2 wt.% from polymers) and sorbitol as a plasticizer (25 wt.% from polymers). For this purpose, we used the approach of the preliminary activation of the interlayer space of the initial bentonite clay particles in water for a long time, followed by the mechanical capture and intercalation of dissolved GEO from a mixture of water and ethanol in the interlayer space of the particles without using surface-active and toxic agents; after that, a lyophilization technique was used. This method demonstrated the efficiency of GEO-loading into the filler structure, which was recorded qualitatively by lyophilization, ATR-FTIR spectroscopy, and XRD analysis methods and quantitatively by thermogravimetry. Using the laser analysis of filler dispersions, it was found that the encapsulation using mechanical methods produced two statistical ensembles of filler particles: small exfoliated particles with an average diameter of less than 1 μm and large intercalated particles with diameters of 5–20 μm. In addition, XRD analysis showed an increase in the d-spacing (3–5%) in the filler particles, which also demonstrated the efficiency of activation and GEO-loading. Also, it was shown that bentonite particles filled with GEO formed a stable dispersion in the water, even when they were centrifuged, presumably due to a decrease in their density, which allowed them to be used to obtain a highly dispersed biocomposite (filler) in a polymer matrix. Optical and scanning electron microscopy of films based on a chitosan/cassava starch mixture in a ratio of 80/20 wt.% filled with bentonite clay/GEO particles showed a high degree of dispersion of particles in the polymer matrix without their coagulation. Moreover, EDS maps confirmed the presence of filler microparticles in the structure of the films. The results of mechanical tests of biocomposite films under tension with different filler contents showed an increase of 40% for tensile strength, 19% for Young’s modulus, and 44% for elongation at break with the introduction of the filler and an increase in its concentration to 1 wt.% in the composition due to the formation of a dense reinforcing network. At the same time, an increase in the filler concentration to 2 wt.% led to a decrease in these properties of the films due to the formation of stress areas. The presence of GEO components in the structure of the filler particles made it possible to increase the antibacterial activity of the original film with respect to *E. coli* and *S. aureus* microorganisms, which indicates an effective release of GEO from the particle structure and inhibition of the growth zone of microorganisms.

## Figures and Tables

**Table 1:** The values of 2*θ* angle and corresponding calculated d-spacing values for bentonite clay particles after their activation in water and middle fraction of the dispersion of bentonite clay/GEO particles.

Sample	2*θ* (Degree)	d-Spacing (Å)	Δ d-Spacing (Å)
Activated bentonite clay	19.9	4.52	0.16
	29.0	3.18	0.09
	35.0	2.68	0.08
	43.3	2.25	0.10
Middle fraction	19.8	4.55	0.19
	28.8	3.20	0.11
	35.0	2.68	0.08
	42.9	2.26	0.11

Δ d-spacing = d-spacing of the sample − d-spacing value from literature [44,51].

## Data Availability

Data are contained within the article.
